# Murine Aortic Smooth Muscle Cells Acquire, Though Fail to Present Exogenous Protein Antigens on Major Histocompatibility Complex Class II Molecules

**DOI:** 10.1155/2014/949845

**Published:** 2014-07-20

**Authors:** Marcella Maddaluno, Neil MacRitchie, Gianluca Grassia, Armando Ialenti, John P. Butcher, Paul Garside, James M. Brewer, Pasquale Maffia

**Affiliations:** ^1^Department of Pharmacy, University of Naples Federico II, 80131 Naples, Italy; ^2^Centre for Immunobiology, Institute of Infection, Immunity and Inflammation, College of Medical, Veterinary and Life Sciences, University of Glasgow, 120 University Place, Glasgow G12 8TA, UK; ^3^Novartis Vaccines, Via Fiorentina 1, 53100 Siena, Italy

## Abstract

In the present study aortic murine smooth muscle cell (SMC) antigen presentation capacity was evaluated using the E*α*-GFP/Y-Ae system to visualize antigen uptake through a GFP tag and tracking of E*α* peptide/MHCII presentation using the Y-Ae Ab. Stimulation with IFN-*γ* (100 ng/mL) for 72 h caused a significant (*P* < 0.01) increase in the percentage of MHC class II positive SMCs, compared with unstimulated cells. Treatment with E*α*-GFP (100 *μ*g/mL) for 48 h induced a significant (*P* < 0.05) increase in the percentage of GFP positive SMCs while it did not affect the percentage of Y-Ae positive cells, being indicative of antigen uptake without its presentation in the context of MHC class II. After IFN-*γ*-stimulation, ovalbumin- (OVA, 1 mg/mL) or OVA_323–339_ peptide-(0.5 *μ*g/mL) treated SMCs failed to induce OT-II CD4^+^ T cell activation/proliferation; this was also accompanied by a lack of expression of key costimulatory molecules (OX40L, CD40, CD70, and CD86) on SMCs. Finally, OVA-treated SMCs failed to induce DO11.10-GFP hybridoma activation, a process independent of costimulation. Our results demonstrate that while murine primary aortic SMCs express MHC class II and can acquire exogenous antigens, they fail to activate T cells through a failure in antigen presentation and a lack of costimulatory molecule expression.

## 1. Introduction

Atherosclerosis is an immunoinflammatory process [1, 2] in which smooth muscle cells (SMCs) play a critical role [3–5]. SMCs produce a broad range of immunoinflammatory mediators contributing to vascular inflammation [6] and participate in the formation of arterial tertiary lymphoid tissue in experimental atherosclerosis [7]. Human SMCs express class II major histocompatibility complex molecules (MHC class II) in atherosclerotic plaques [8] and following IFN-*γ* stimulation [9–11]. In addition, SMC MHC class II expression increases following vascular injury in rodent models [12]. However, the possibility that SMCs can act as antigen presenting cells (APCs) and consequently activate vascular T cell response remains, to date, controversial. In mice it has been demonstrated that brain microvessel SMCs/pericytes can induce a proliferation of syngenic CD4^+^ T cells* in vitro* in a MHC class II dependent manner [13]. SMCs/pericytes were able to process and present exogenous antigens to T cell hybridoma [14] and preferentially activated Th1 T cell clones as compared with Th2 T cells of the same antigen specificity [15]. In contrast to syngeneic cocultures using wild type CD4^+^ T cells, microvascular SMCs did not support proliferation of antigen specific T cell receptor (TCR) transgenic CD4^+^ T cells [16]. Others demonstrated that murine SMCs pulsed with antigen increased the expression of the IL-2 receptor on T cells but were not able to induce T cell proliferation [17].

Human saphenous vein SMCs expressing MHC class II molecules were unable to activate allogeneic memory T cells [18] and failed to effectively support T cell proliferation to the polyclonal activator, phytohemagglutinin [19]. This inability resulted from a defect in costimulatory function, particularly the lack of OX40 ligand (OX40L) [19]. SMCs from different tissues may behave differently; for example, cultured human airway smooth muscle cells were capable of presenting the superantigen, staphylococcal enterotoxin A, via MHC class II molecules to CD4^+^ T cells [20]. More selective approaches are required to investigate SMC antigen presentation capacity.

Here we utilized the E*α*-GFP/Y-Ae model that allows visualization of antigen uptake through a GFP tagged E*α* peptide and tracking of antigen presentation using the Y-Ae Ab. The E*α*-GFP protein is internalized and processed by APCs to generate E*α* peptide for presentation on MHC class II. The monoclonal Ab Y-Ae detects E*α* only when bound to MHC class II molecules (I-A^b^) [21–24]. We demonstrate that while murine primary aortic SMCs express MHC class II and can acquire exogenous antigens, they fail to activate T cells through a failure in antigen presentation and a lack of costimulatory molecule expression.

## 2. Materials and Methods

### 2.1. Animals

C57BL/6 mice (Harlan, Shardlow, UK) were used to prepare SMCs and dendritic cells (DCs). OT-II (CD45.1) mice bred in house were used as donors of Tg T cells. These transgenic mice express the mouse alpha-chain and beta-chain T cell receptor that pairs with the CD4 coreceptor and is specific for chicken ovalbumin 323–339 in the context of I-A^b^. Animals were maintained on a 12/12-hour light/dark cycle with free access to food and water and all the procedures were performed in accordance with local ethical and UK Home Office regulations.

### 2.2. Cell Cultures and Cocultures

Murine primary SMCs were derived from the thoracic aorta of C57BL/6 mice as previously described [25, 26] and grown in DMEM supplemented with L-glutamine, 10% fetal bovine serum, 100 U/mL penicillin, and 100 *µ*g/mL streptomycin (all from Gibco, Paisley, UK). Before initiation of the assays, the SMCs were starved into DMEM supplemented with 0.1% fetal bovine serum for 48 hours [25, 27]. Cells were characterized by immunofluorescence microscopy using FITC labeled anti-smooth muscle *α*-actin (*α*-SMA) monoclonal antibody (Ab) (clone 1A4; Sigma-Aldrich, Dorset, UK). Studies were performed with cells at passages 3–6. OVA specific TCR transgenic OT-II CD4^+^ T cells were isolated from OT-II/CD45.1 mice using the MicroBead-based CD4^+^ T Cell Isolation Kit II (Miltenyi Biotec, Bisley, UK) according to manufacturer's instructions and grown in complete RPMI (containing L-glutamine, 10% fetal bovine serum, 100 U/mL penicillin, and 100 *µ*g/mL streptomycin). The DO11.10-GFP hybridoma cells [28] were grown in complete RPMI containing geneticin (0.5 mg/mL, Sigma-Aldrich) as previously described [29]. DCs were obtained by flushing the bone marrow of C57BL/6 mice and grown in complete RPMI containing 10% granulocyte-macrophage colony stimulating factor (GM-CSF) for 7 days [30]. All cells used were kept in a humidified incubator at 37°C in 5% CO_2_.

Murine SMCs were cultured in 48 multiwell plates until 80% confluence. Subsequently cells were stimulated with IFN-*γ* (100 ng/mL; R&D Systems, Abingdon, UK) for 72 h to enhance their MHC class II expression and then treated with OVA (1 mg/mL; InvivoGen, Toulouse, France) or OVA_323–339_ peptide (0.5 *µ*g/mL; InvivoGen) overnight. Isolated OT-II CD4^+^ T cell or DO11.10-GFP hybridoma cell preparations were then introduced into the murine SMC cultures at a 1 : 5 ratio, for 24, 48, and 72 h or 24 h, respectively. OVA-treated DCs, cocultured with both OT-II CD4^+^ T cells and DO11.10-GFP hybridoma cells at the same ratio of SMCs, were used as positive control. Subsequently, OT-II CD4^+^T cells or DO11.10-GFP hybridoma cells were collected by rinsing the cocultures three times followed by staining and preparation for flow cytometric analysis. For the analysis of costimulatory molecule expression murine SMCs were cultured in 6 multiwell plates and stimulated with IFN-*γ* (100 ng/mL) for 72 h before flow cytometry. In a separate set of experiments, SMCs were stimulated with IFN-*γ* (100 ng/mL) for 72 h and then treated with fluorescein labeled-chicken OVA (FITC-OVA, 1 mg/mL, Molecular Probes) overnight. Subsequently, the supernatant was removed and the cells washed with PBS. The FITC-OVA uptake was visualized using the EVOS FL Cell Imaging System (Life Technologies Ltd., Paisley, UK).

### 2.3. Ealpha-GFP Preparation and Treatment

To assess the ability of murine SMCs to act as APCs, we employed the Ealpha- (E*α*-) GFP/Y-Ae system as previously described [22–24]. A recombinant* Escherichia coli* strain expressing the E*α*-GFP fusion protein was grown to midlog phase before induction of protein expression. Protein expression was induced by addition of isopropyl *β*-D-1-thiogalactopyranoside (IPTG; Sigma-Aldrich) to a final concentration of 1 mM and cultures were incubated overnight at 30°C with agitation (200 rpm). The E*α*-GFP fusion protein was purified from the bacterial lysates using HisPur Cobalt Spin Columns (Thermo Scientific, Loughborough, UK) and endotoxin was removed using Detoxi-Gel Endotoxin Removing Columns (Thermo Scientific). Murine SMCs were cultured in 6 multiwell plates, as described above, stimulated with IFN-*γ* (100 ng/mL) for 72 h, and then treated with E*α*-GFP (100 *µ*g/mL). After 1, 24, and 48 h of treatment, cells were collected for flow cytometric analysis. DCs cultured under the same conditions and treated with E*α*-GFP (100 *µ*g/mL) for 24 h were used as a positive control.

### 2.4. Flow Cytometry

Aliquots of cells were washed and resuspended in Fc block (2.4G2 hybridoma supernatant) for 25 mins at 4°C to block Fc receptors. Subsequently, cells were incubated with Abs (in PBS containing 2% FBS) for 30 mins at 4°C, washed twice and then, where necessary, incubated with streptavidin for additional 20 mins at 4°C. Following washing, cells were analyzed on a FACScalibur using CellQuest-Pro (BD Biosciences, Oxford, UK), or on a MACSQuant Analyzer (Miltenyi Biotec). Data analysis was performed using 6 FlowJo (Tree Star Inc., Olten, Switzerland).

Murine SMCs were stained with the following primary Abs: Y-Ae-Bio (specific for I-E*α* 52–68 presented on I-A^b^; clone: eBioY-Ae), anti-MHC II (I-A/I-E)-APC (clone: M5/114.15.2), anti-CD11c-APC (clone: N418), anti-CD54-PE (clone: 3E2), anti-CD44-FITC (clone: IM7), anti-OX40L-Bio (clone: RM134L) followed by streptavidin-PerCP, anti-CD80-FITC (clone: 16-10A1), anti-CD40-PE (clone: 3/23), anti-CD86-APC (clone: GL1), and anti-CD70-Bio (clone: FR70) followed by streptavidin-PerCP. OT-II CD4^+^ T cells were stained with primary mAbs anti-CD4-PerCP (clone: RM4-5), anti-CD25-APC (clone: PC61), anti-CD44-PE (clone: IM7), and anti-CD69-Bio (clone: H1.2F3) followed by streptavidin-Pacific Blue. DO11.10-GFP hybridoma cells were stained with the primary Ab anti-DO11.10 TCR-APC (clone: KJ 1-26). Isotype-matched Abs were used as negative control. Y-Ae Ab, anti-CD11c, and anti-MHC II Ab were from eBioscience (Hatfield, UK); streptavidin-Pacific Blue was from Life Technologies Ltd.; all other Abs were from BD Biosciences.

### 2.5. CFSE Staining

OT-II CD4^+^ T cells were labeled with the fluorescent dye carboxyl fluorescein succinimidyl ester (CFSE, Molecular Probes) as previously described [31]. The cells were washed and then cocultured with SMCs or DCs (used as a positive control) for 72 h. The level of fluorescence intensity from the CFSE labeling was measured by flow cytometry. Incremental loss of CFSE intensity showed proliferation.

### 2.6. Statistical Analysis

Results are expressed as mean ± SEM of 3 experiments run in triplicate. The results were statistically analyzed by the *t*-test or ANOVA (Two-Tail *P* value) and the Bonferroni post hoc test. The level of statistical significance was *P* < 0.05 per test.

## 3. Results

### 3.1. Assessment of Antigen Uptake/Presentation by SMCs Using the E*α*-GFP/Y-Ae System

**Figure 1:**
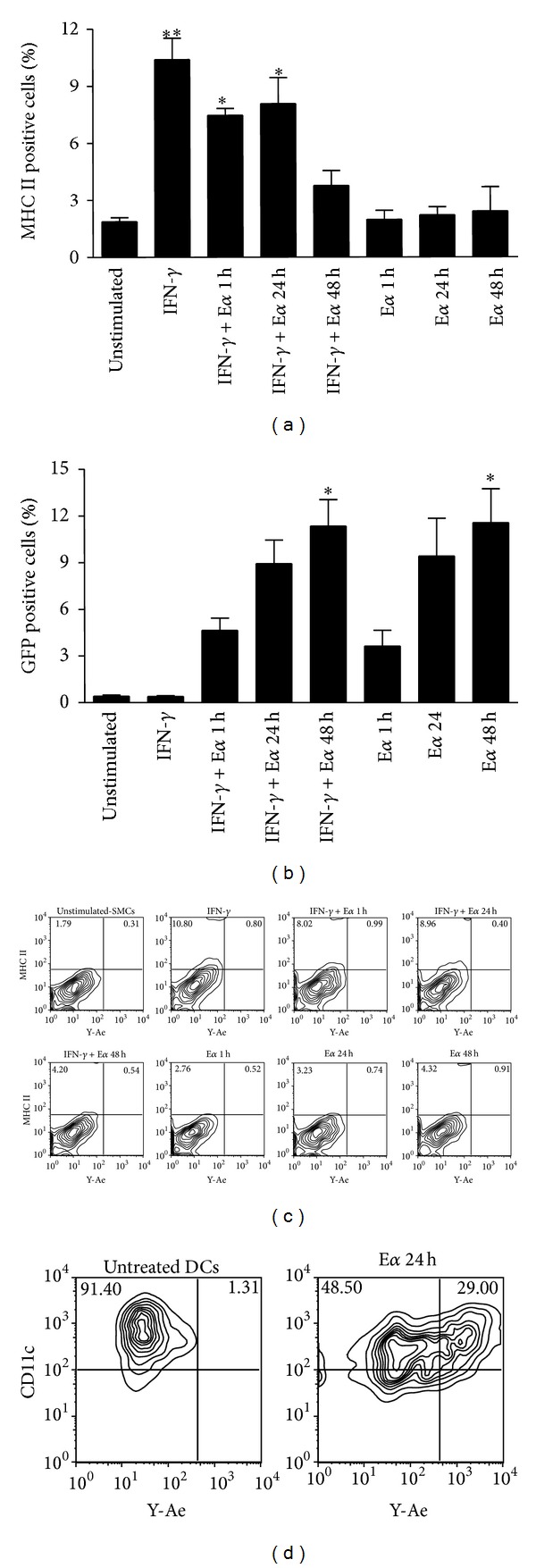
SMCs acquire exogenous antigens but fail to present them in the context of MHC class II. Evaluation of antigen uptake/presentation by murine SMCs. SMCs were stimulated with IFN-*γ* (100 ng/mL) for 72 h and subsequently treated with E*α*-GFP peptide (100 *µ*g/mL) for the indicated time points. (a) MHC class II expression. (b) GFP expression. (c) Representative flow cytometry plots showing no positivity of SMCs to the Y-Ae Ab or (d) positivity of DCs, used as a positive control. Results are expressed as mean ± SEM from three separate experiments. *P < 0.05, **P < 0.01, versus unstimulated cells.

Stimulation with IFN-*γ* (100 ng/mL) for 72 h resulted in a significant (*P* < 0.01) 5- to 6-fold increase in the percentage of MHC class II positive SMCs compared with unstimulated cells (Figure 1(a)). Similar results were observed in IFN-*γ*-stimulated SMCs subsequently treated with E*α* peptide (100 *µ*g/mL) for 1 and 24 h (*P* < 0.05), while no significant changes were observed after 48 h of treatment (Figure 1(a)). As shown in Figure 1(b), SMC treatment with E*α* peptide induced an increase in the percentage of GFP positive cells, both in presence or absence of IFN-*γ*-stimulation, being indicative of antigen uptake. The increase in GFP positive cells observed was significant only at 48 h (*P* < 0.05). No significant changes were observed in the percentage of Y-Ae positive SMCs after IFN-*γ*-stimulation and/or treatment with E*α* peptide (Figure 1(c)) suggesting that, although SMCs internalize the antigen, they are not able to present the E*α* peptide in the context of MHC class II. Treatment of DCs with E*α* peptide (100 *µ*g/mL), used as positive control, caused an increase in the percentage of Y-Ae positive cells (Figure 1(d)).

### 3.2. SMCs Fail to Induce OT-II CD4^+^ T Cell Activation and Proliferation

**Figure 2:**
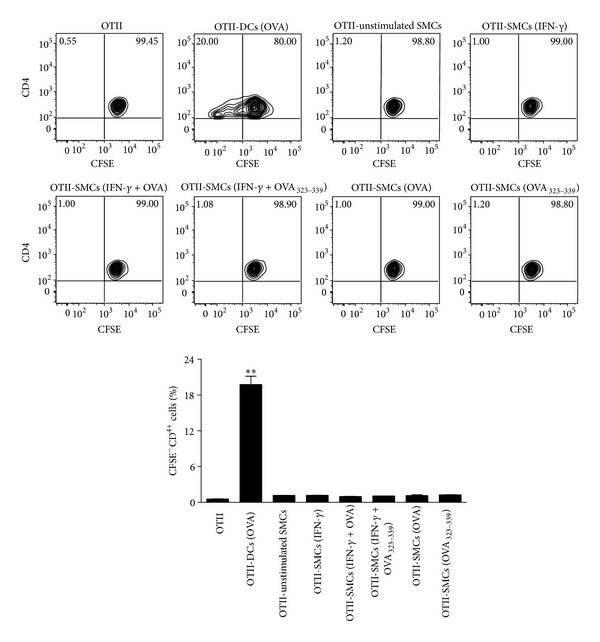
SMCs fail to induce OT-II CD4^+^ T cell proliferation. Representative plots and relative statistical analysis showing the effect of SMCs on OT-II CD4^+^ T cell proliferation. IFN-*γ*-stimulated SMCs were treated with OVA (1 mg/mL) or OVA_323–339_ peptide (0.5 *µ*g/mL) overnight and then cocultured with CFSE-labeled OT-II CD4^+^ T cells for 72 h. OVA-treated DCs were used as a positive control. Results are expressed as mean ± SEM from three separate experiments run in triplicate. **P < 0.01 versus OT-II CD4^+^ T cells alone.

We next assessed the ability of SMCs to activate OVA-specific transgenic CD4^+^ T cells. In preliminary experiments by using FITC-OVA we confirmed the uptake of the model antigen by SMCs (data not shown). Using CFSE to track proliferation, we evaluated the number of Tg T cells undergoing proliferation after 72 h of coculture with SMCs or bone marrow derived DCs, used as positive control. The proportion of dividing T cells (expressed as percentage of CFSE^−^ CD4^+^ cells) was approximately 0.5–1% in both presence and absence of cocultured unstimulated SMCs (Figure 2). Neither stimulation with IFN-*γ* nor treatment with OVA or OVA_323–339_ peptide of SMCs affected the proliferation of OT-II CD4^+^ T cells. In contrast, coculture with OVA-treated DCs significantly (*P* < 0.01) increased the proportion of dividing OT-II CD4^+^T cells by around 20% (Figure 2).

**Figure 3:**
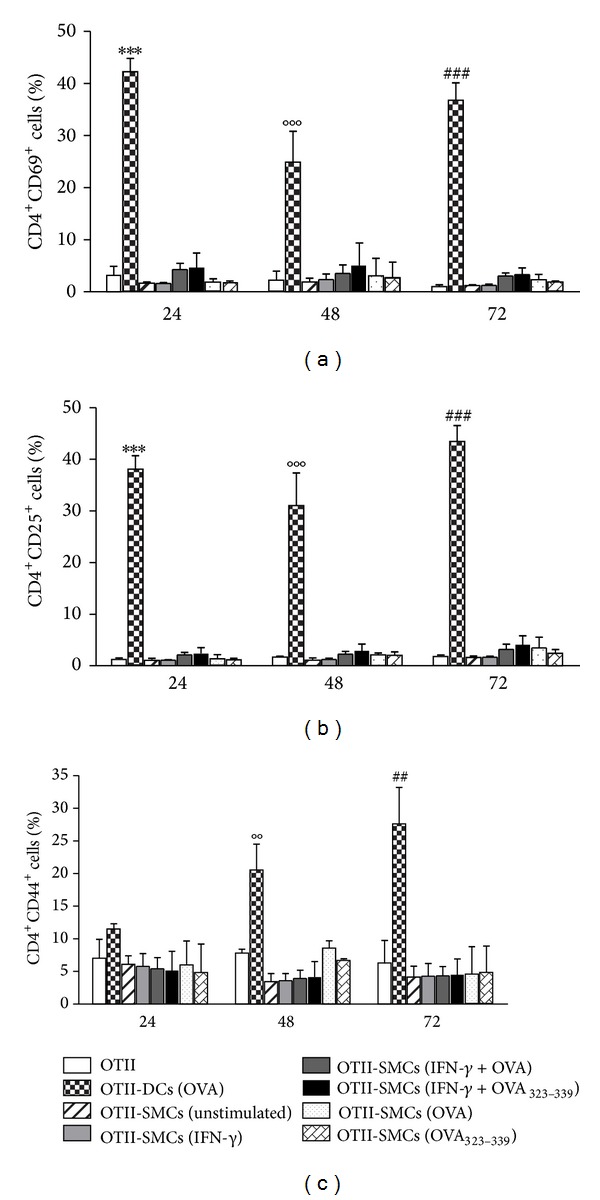
SMCs fail to induce OT-II CD4^+^ T cell activation. Expression of CD69, CD25, and CD44 on OT-II CD4^+^ T cells cocultured with OVA- or OVA_323–339_ peptide-treated SMCs or OVA-treated DCs (used as positive control). Results are expressed as mean ± SEM from three separate experiments run in triplicate. ***P < 0.001 versus OT-II CD4^+^ T cells alone at 24 h; °°*P* < 0.01, °°°*P* < 0.001 versus OT-II CD4^+^ T cells alone at 48 h; ^##^ *P* < 0.01 and ^###^ *P* < 0.001 versus OT-II CD4^+^ T cells alone at 72 h.

We also examined cell surface expression of activation markers such as CD25, CD44, and CD69 on OT-II CD4^+^ T cells after coculture with SMCs or bone marrow derived DCs. CD25 and CD69 were detected in approximately 2% of OT-II CD4^+^ T cells, alone or cocultured for 24, 48, and 72 h with unstimulated SMCs, IFN-*γ*-stimulated SMCs, or IFN-*γ*-stimulated SMCs treated with OVA or OVA_323–339_ peptide. Moreover, the percentage of CD25 and CD69 positive T cells did not change after SMC treatment with OVA or OVA_323–339_ peptide alone, while a significant (*P* < 0.001) increase was observed only after coculture with OVA-treated DCs at all of the time points considered (Figure 3). The percentage of CD44 positive OT-II CD4^+^ T cells was about 7% at all of the time points considered, in both presence and absence of unstimulated SMCs. Stimulation with IFN-*γ* and/or treatment of SMCs with OVA or OVA_323–339_ did not affect CD44 expression. A significant (*P* < 0.01) increase in CD44 positive OT-II CD4^+^ T cells was observed after 48 and 72 h of coculture with OVA-treated DCs (Figure 3). These data demonstrate that antigen-pulsed aortic murine SMCs are not able to induce antigen-specific T cell activation/proliferation.

### 3.3. Effect of IFN-*γ* Stimulation on Costimulatory/Adhesion Molecules Expression by Murine SMCs

**Figure 4:**
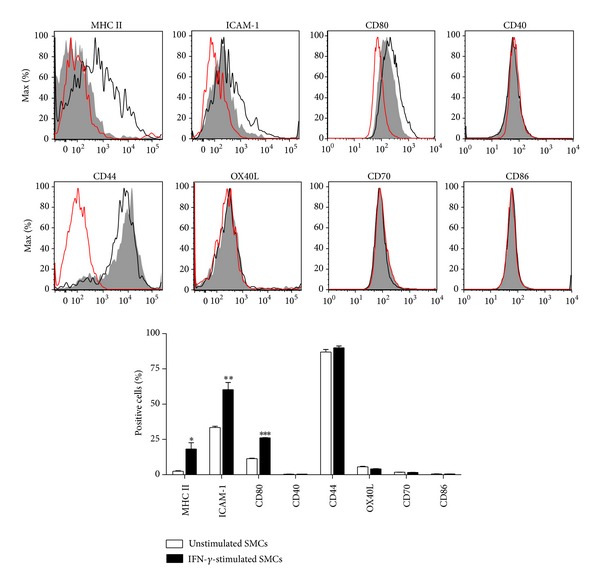
SMCs lack key costimulatory molecules. Representative flow cytometry histograms and relative graph showing the effect of IFN-*γ* (100 ng/mL) on costimulatory/adhesion molecules expression in murine SMCs. Red empty histograms: isotype control; gray filled histograms or white columns: unstimulated SMCs; black empty histograms or black columns: IFN-*γ*-stimulated SMCs. Results are expressed as mean ± SEM from three separate experiments run in triplicate. *P < 0.05, **P < 0.01, and ***P < 0.001 versus unstimulated cells.

Previous studies have correlated the inability of human SMCs to activate memory T cells with the lack of costimulation [19]. Thus we examined whether murine SMCs express costimulatory/adhesion molecules at baseline and after IFN-*γ* (100 ng/mL) stimulation for 72 h. As shown in Figure 4, unstimulated SMCs expressed CD54 (ICAM-1), CD80, and CD44 (30%, 11%, and 87% positive cells, resp.). The stimulation with IFN-*γ* caused a 2-fold increase in the percentage of both ICAM-1 (*P* < 0.01) and CD80 (*P* < 0.001) positive cells while it did not affect the percentage of CD44 positive cells. In contrast, only low levels of OX40L, CD40, CD70, and CD86 expression were detectable in unstimulated SMCs. IFN-*γ* stimulation did not increase the percentage of SMCs positive to these molecules. The failure of SMCs to respond to IFN-*γ*, in this case, was selective for the costimulatory molecules because the percentage of MHC class II molecules was increased after IFN-*γ* stimulation under the same conditions (Figure 4).

### 3.4. SMCs Do Not Activate DO11.10-GFP Hybridoma Cells

**Figure 5:**
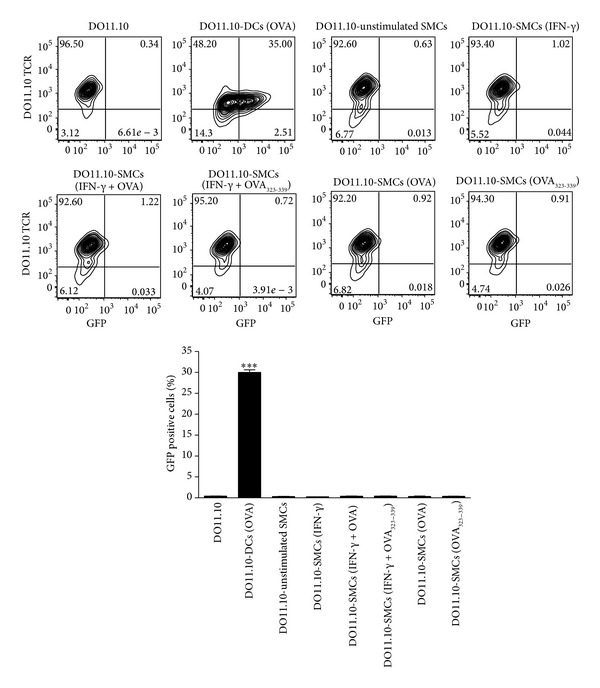
Effect of SMCs on DO11.10-GFP hybridoma cell activation. IFN-*γ*-stimulated SMCs were treated with (OVA, 1 mg/mL) or OVA_323–339_ peptide (0.5 *µ*g/mL) overnight and then cocultured with DO11.10-GFP hybridoma cells for 24 h. OVA-treated DCs were used as positive control. Results are expressed as mean ± SEM from three separate experiments run in triplicate. ***P < 0.001 versus DO11.10-GFP hybridoma cells alone.

The murine DO11.10-GFP hybridoma was originally obtained by stably transfecting a DO11.10 T cell hybridoma with a construct in which GFP expression is under the control of a nuclear factor of activated T cells (NFAT) regulated promoter [28]. Thus, once activated, hybridoma cells, detectable using the KJ1-26 clonotypic antibody, become GFP-positive. DO11.10 hybridoma cells express the TCR recognizing OVA_323–339_ peptide in the context of either I-A^d^ or I-A^b^ MHC class II [32] without any requirement for costimulation [29]. Coculture with unstimulated SMCs had no effect on GFP expression by DO11.10-GFP hybridoma cells and similar results were obtained after stimulation with IFN-*γ* and/or treatment of SMCs with OVA or OVA_323–339_ peptide. On the contrary, DCs treated with OVA, used as positive control, caused a significant (*P* < 0.001) increase in GFP expression by hybridoma cells (Figure 5). These data confirm that SMCs are unable to present exogenous protein antigens in the context of MHC class II.

## 4. Discussion

In the present study, we demonstrated that (1) cultured primary murine SMCs express MHC class II molecules after stimulation with IFN-*γ* and are able to acquire/uptake antigens; however, they fail to present the peptide antigen in the context of MHC class II, as demonstrated by using the specific Ealpha- (E*α*-) GFP/Y-Ae system; (2) OVA-treated SMCs fail to induce activation/proliferation of OT-II CD4^+^ T cells, data consistent with a defect in MHC class II-restricted Ag presentation and in the expression of costimulatory molecules, such as OX40L, CD40, CD70, and CD86; (3) SMCs also fail to promote activation of OVA responding DO11.10-GFP hybridoma T cells that do not require any costimulatory signal for activation.

The first finding that murine aortic SMCs express MHC class II molecules is in line with previous data showing MHC class II expression in atheroma SMCs [8] and in rodent arteries in response to injury [12], as well as in human SMCs in culture following IFN-*γ*-stimulation [18]. Murray and colleagues [18] demonstrated that class II molecules on human saphenous vein SMCs were functional, since they induced CD25 expression on resting CD4^+^ T cells. Additional studies demonstrated that survival and activation of T cells occurred as a result of the specific interaction between TCR on T cells and MHC molecules on SMCs, since treatment with antibodies directed toward MHC class II blocked the proliferation of CD4^+^ T cells cocultured with syngeneic SMCs [13, 16]. On the contrary, in the context of nonspecific generalized T cell stimulation or in the presence of polyclonal activators such as phytohemagglutinin SMCs did not activate CD4^+^ T cells [18, 19].

In order to understand whether an antigen specific stimulation leads to immunological competence of SMCs, engaging MHC molecules, we employed a novel and selective approach such as the E*α*-GFP/Y-Ae model that allows visualization of antigen uptake through a GFP tagged E*α* peptide and tracking of antigen presentation using the Y-Ae Ab. The E*α*-GFP protein is internalized and processed by APCs to generate E*α* peptide for presentation on MHC class II. The monoclonal Ab Y-Ae detects E*α* only when bound to MHC class II molecules (I-A^b^) [21–24]. E*α*-GFP treatment of SMCs increased the percentage of GFP positive cells, without affecting the percentage of SMCs positive to the monoclonal Ab Y-Ae. These results clearly demonstrate that primary aortic murine SMCs fail to present exogenous protein antigens in the context of MHC class II.

Our results also prove the inability of SMCs in inducing OVA specific OT-II CD4^+^ T cell activation and proliferation. A possible explanation for these observations is that SMCs fail to activate T cells through a failure in antigen presentation and a lack of costimulatory molecule expression. Indeed, although human SMCs express the costimulatory molecules CD44, CD54, CD58, and CD59 [18], they lack OX40L, which is considered essential for T cell activation [19]. We also observed lack of costimulatory molecule expression (OX40L, CD40, CD70, and CD86) on SMC surface following IFN-*γ* stimulation, supporting the hypothesis that impaired costimulation function contributes to the inability of SMCs to induce T cells activation/proliferation.

In order to analyze this point further, we cocultured SMCs with the DO11.10-GFP hybridoma cells that, in presence of the model Ag OVA, undergo activation without requiring any costimulatory signal [28, 29]. Importantly, SMCs failed to activate DO11.10-GFP hybridoma cells, demonstrating that other mechanisms, apart from a defect in costimulation function, are liable for the limited capacity of SMCs to activate T cells.

One possibility could be that SMCs cannot process protein antigens, rather than not being able to present them. Nevertheless, in our experiments, treatment of SMCs with OVA_323–339_ peptide, which does not require any processing to be presented in the context of MHC molecules, did not affect activation/proliferation of neither OT-II CD4^+^ T cell nor DO11.10-GFP hybridoma cells. This observation demonstrates that the SMC inability in presentation cannot lie in a defect in the antigen processing; thus further investigations will be necessary to understand the mechanisms underlining this deficiency.

## 5. Conclusions

In summary, our work demonstrates that while murine primary aortic SMCs express MHC class II and can acquire exogenous antigens, they fail to activate T cells through a failure in antigen presentation and a lack of costimulatory molecule expression. Our results do not preclude the possibility that SMCs could act as APCs, depending on the environment (e.g., in atherosclerotic arteries) and the vascular bed; however, they suggest that antigen presentation may not be the key immunological feature of SMCs in the initiation of vascular inflammation.
